# Digitalisation of medicine in LMICs (sub-Saharan Africa): enablers, barriers, and funding strategies for sustainable health systems

**DOI:** 10.3389/fpubh.2026.1710904

**Published:** 2026-02-27

**Authors:** Sandra Nanyonga, Peter Blanshard, Lisa Maria Crevola, Daniel Simeon-Dubach

**Affiliations:** 1 Université Côte d'Azur, Nice, France; 2 Independent Consultant, Amsterdam, Netherlands; 3 Johnson & Johnson, Zug, Switzerland; 4 Medservice, Biobank & Biobanking Consulting and Services, Walchwil, Switzerland

**Keywords:** digitalisation of medicine, funding, incentives, investments, LMICs, sub-Saharan Africa

## Abstract

For the vast majority of low- and middle-income countries (LMICs), universal health coverage is very difficult to achieve for a variety of reasons. Digitalisation of healthcare is promoted as a way to expand access and strengthen health systems, yet in many LMICs, including those in sub-Saharan Africa, digital initiatives remain fragmented, poorly coordinated, and underfunded. This article explores how digital health technologies can support outbreak preparedness, routine service delivery, and data-driven decision-making, and outlines the ethical, legal, social, and financial considerations that shape their sustainable adoption. Using real-world examples such as digital tools for infectious disease surveillance, biobanking and data governance models, and national digital health strategies, it highlights both the potential of digitalisation and the risk of widening inequities when infrastructure, regulation, and local ownership are weak. The article argues that digital health investments must align with country priorities, be embedded in robust governance frameworks, and be supported by long-term financing that balances public, private, and donor contributions to enable more coordinated and equitable implementation in LMICs.

## 1 Introduction

Digitalisation is often uncritically hailed as a universal solution to many problems in healthcare. Indeed, there are numerous examples where digitalisation has improved or simplified access to healthcare, alleviated strains due to lack of healthcare workers, and enabled the management of emerging diseases. Despite the enthusiasm for digital health, implementation has experienced challenges including an escalation of short-lived digital health interventions, duplication, and minimal documentation of evidence on their impact on the health system (1).

Financial considerations always play a decisive role in the design and subsequent scaling of such projects. However, decision-makers should be aware that in health systems where cost pressure is high, investments in digital infrastructure are particularly crucial. The United Nations Development Programme (UNDP) sees digital technology as a key factor that will help expand universal health coverage by eliminating a number of barriers such as prohibitively high costs, complicated access and lack of quality of care, while extending the range of services, particularly in regions where infrastructure and personnel are scarce or non-existent (2).

There is an urgent need to address both emerging and long-standing healthcare challenges with digital solutions. Only by realising the full potential of digital health can the sustainable development goal 3 (SDG 3) “Good health and well-being for all” be achieved. This article shows how investments in digital health are worthwhile, deals with the question of financing and shows how such projects can be sustainably implemented and scaled. Ultimately, digital health needs to be put at the service of policy makers, healthcare professionals and, first and foremost, the general population and patients.

This paper provides a concise synopsis of key points pertaining to digitalisation in LMICs. This is founded upon our own professional experience, which is the rationale behind our deliberate focus on LMICs in sub-Saharan countries. First, we address general and non-financial aspects of digital health adoption in LMICs, illustrated through case studies. Next, we discuss ethical, legal, and social challenges specific to these settings. Finally, we present a financial perspective by highlighting key financing requirements, challenges and an overview of available funding sources.

## 2 Context and real-world examples

### 2.1 Digital solutions for managing infectious disease outbreaks in sub-Saharan Africa

Centred around the global strategy on digital health 2020–2025, the World Health Organization (WHO) is pursuing a vision of improving health for everyone through digital health solutions including smart and connected devices, the Internet of Things, advanced computing, big data analytics, artificial intelligence (AI), and robotics. Digital health technologies are an important component for national strategies, but their implementation can be challenging, especially in LMICs (3, 4). Healthcare challenges in LMICs are diverse and amongst others include limited funding, technology and skills, low quality and standards, weak policy frameworks, small markets, unstable demand and poor infrastructure (5). Issues regarding the funding of health systems are particularly critical. This is also true in Africa where most countries have still not met the goals of the 2001 Abuja Declaration, in which governments pledged to devote 15 percent of their national budgets to health. Health systems across Sub-Saharan Africa face multiple challenges and service gaps, which occur at varying levels of severity but are present in every country in the region (2). At the same time, however, we see that with the rapid expansion and evolution of digital solutions, sub-Saharan Africa has become a powerhouse for digital health innovations directed at strengthening healthcare with respect to patient management, disease surveillance and prevention. E-Health strategies are being developed and implemented at impressive speed. Forty-one out of fifty-four African countries have national digital health strategies and architectures (6). As the traditional health-care systems are typically ill-equipped, understaffed, or not accessible at all, sub-Saharan Africa is booming with digital solutions to overcome these structural problems (6).

In many respects, sub-Saharan Africa is closing the digital gap with the rest of the world. Internet penetration is expanding rapidly, especially through mobile connectivity. Indeed, some countries in the region—Cabo Verde, Ghana, Rwanda, and the Seychelles—are leaders in their income group. Many sub-Saharan African countries deployed digital policy responses to cope with, and cushion the effects of, the COVID-19 pandemic (7). In Rwanda, for example, anti-epidemic robots monitored patients, delivered food and medication, and kept medical records. Designed with various advanced features, the robots supported doctors and nurses at the designated treatment centres (8). To implement cost-efficient testing strategies with rapid turnaround and community-based contact-tracing, sub-Saharan African countries had to capitalise on digital health innovations such as mobile phone technology (mHealth) platforms. Rwanda and South Africa implemented best practices that include digital contact tracing with the use of a mobile app and cell phone tower data, respectively, hence reducing the required workforce (9). The COVID-19 response in South Africa has shown that a robust healthcare service platform, which includes laboratory testing, hospital bed space, and community engagement, sensitisation, and involvement in contact tracing, is essential. Best practices for contact tracing further included the deployment of community health workers and intense involvement of public health specialists to ensure that different aspects of the response are appropriately managed, including identification of contacts, epidemiology, and surveillance, as well as updating central electronic databases. A user-friendly, centralised database within a network that fed-in data from both private and public sectors was critical to contact tracing, case finding, and overall surveillance (9).

### 2.2 Handling viral outbreaks as an example of the usefulness of digital health

The coronavirus outbreaks (SARS-CoV-2, SARS-CoV and MERS-CoV) and other viral outbreaks such as Zika and Ebola virus show that infectious diseases should be considered among the most important health hazards and challenges that we will face in the foreseeable future. The recurring viral outbreaks serve as reminders that proactive planning for healthcare emergencies as well as intensified commitment to global public health preparedness remains necessary (10). COVID-19 and Ebola are very different diseases, but they triggered similar public health responses, therefore, the lessons made from the 2014–2016 Ebola crisis are still relevant today. The Ebola outbreaks across West Africa resulted in significant human fatalities and had a huge impact on lives and livelihoods of residents of countries such as Guinea, Liberia, and Sierra Leone, where major outbreaks occurred. Those Ebola outbreaks led to the WHO declaration of a Public Health Emergency of International Concern in 2014 that was later ended in 2016, albeit, with many cases subsequently recorded and attributed to flare-ups (11).

#### 2.2.1 Surveillance, data sharing, and digital technologies in outbreak response

These outbreaks revealed the serious shortcomings in national and international capacity to detect, monitor, and respond to infectious outbreaks as they occur. Surveillance, defined as “the systematic ongoing collection, collation and analysis of data for public health purposes and the timely dissemination of public health information for assessment and public health response as necessary” in Article 1 of the International Health Regulations (119), is a critical component of outbreak management. Technological advances in diagnostic tools, genome sequencing, computing power and communications devices can augment traditional surveillance methods to accrue and disseminate information in real time, offering the possibility of better outbreak management and thereby saving lives (12). The failure to collect, store, curate and disseminate data and the low priority for rapid data sharing contributed to a delayed response during the Ebola virus outbreak in West Africa (13). This exemplifies that policies for emergency responses to infectious disease outbreaks are only as good as the data upon which they are based.

Emergency preparedness for public health challenges requires near real-time, broad-based, continuous information, and a collective framework for data collection, sharing, analysis, alerts, investigation, and response. Particularly in the context of LMICs, challenges remain in the infrastructure, not only to collect data, but also to implement data sharing mechanisms. While the burden of disease is more severe in LMICs they often lack adequate infrastructure, workforce capacity, and legal frameworks. Automation and digitisation are central to empowering data use, and technological capacity can be best leveraged when standardised data from many sources are collected and analysed (14). Therefore, there is a definite need in LMICs to replicate the strong international trend towards the development of biobanking infrastructure and facilities, providing means for LMICs inclusion in international research activities (15). The cost of not doing so would be especially great for developing countries where the barriers preventing inclusion in such databases will continue to rise, further excluding these populations and increasing existing biases that favour high-income countries (16).

With resources diverted to fight recurring epidemics, entire health systems essentially shut down, affecting healthcare provision, including diagnostic and treatment of other infectious diseases (17). There is simply insufficient capital investment to support large-scale implementation of health infrastructure projects. In countries with limited resources, interoperability of existing infrastructures can provide relief. During the Ebola outbreak in Sierra Leone residual clinical specimens and accompanying data were collected in the field for routine diagnostic testing in Public Health England (PHE)-led laboratories. PHE operated three diagnostic laboratories in Freetown (Kerrytown), North-West Sierra Leone (Port Loko Laboratory) and Central Sierra Leone (Makeni Laboratory). These laboratories processed up to 300 clinical samples each day during the outbreak. In 2015, approximately 10′000 residual samples and associated data were collected and transferred to PHE in the UK leading to the establishment of the Ministry of Health and Sanitation (MOHS)-PHE Ebola Biobank and the creation of a single governance group, the Ebola Biobank Governance Group (EBGG), to streamline the utilisation of this interoperable infrastructure and facilitate access to samples through this single governance structure.

#### 2.2.2 Governance, equity, and benefit sharing in digital and data-driven research

The EBGG guarantees equality of access to the biobank for the most scientifically valuable research including research from LMICs. Ensuring benefit to the people of Sierra Leone is an overarching principle for decisions of the EBGG. The MOHS in Sierra Leone retains ownership of all the data and materials and is working with PHE and other researchers to develop and conduct a series of research projects that will inform future healthcare and public health strategies relating to Ebola (18, 19). This example illustrates that data standardisation and interoperability of existing infrastructure brings benefits in collaborative research, but it must be strongly emphasised that basic infrastructure should first be in place and that investments must be made in this infrastructure. Without this basic infrastructure, no cooperation is possible, neither under crisis nor under normal circumstances.

During the Ebola virus outbreak, on the 19th and 20th of January 2015, a meeting was held in Dakar (Senegal) to evaluate potential Ebola virus disease treatment options and vaccine candidates for deployment in West Africa. As a result of this Dakar meeting, one recommendation made by the various participants from national health ministries and international organisations, was to strengthen the existing Biosafety Level Containment Facilities (BSL 3/4 laboratories) and to develop professional biobanking infrastructures in Africa to support effective research during episodes of emerging infectious diseases (20). In addition to biobanks, diagnostic laboratories are another essential component of the basic infrastructure of any healthcare system. There is a large gap between diagnostic needs and diagnostic access across much of sub-Saharan Africa, particularly for infectious diseases that inflict a substantial burden of morbidity and mortality. Accurate diagnostics are essential for the correct treatment of individuals and provide vital information underpinning disease surveillance, prevention, and control strategies. Rather than trying to emulate diagnostic laboratory models in resource-rich settings, African countries have the potential to pioneer new models of healthcare designed around digital diagnostics (21). One such project is DIDIDA, or Digital Innovations and Diagnostics for Infectious Diseases in Africa, an EU and UK Innovation funded project developing reliable, low-cost and mobile phone-connected tests to help detect multiple diseases at once in sub-Saharan Africa (22).

### 2.3 From emergency response to national digital health strategies

The Ebola outbreak in West Africa in 2014 exposed serious weaknesses in Sierra Leone’s health information systems (HIS) and health management information systems (HMIS), including delays, incomplete data, and inefficient resource allocation (18, 23). In 2016, WHO worked with the Ministry of Health and Sanitation (MOHS) to plan and prioritise digital systems aligned with national health priorities. This was driven by lessons learned from the fragmented digital health investments during the Ebola response, which highlighted the need for governance structures and strategic planning (24).

In 2017, MOHS launched an eHealth Coordination Hub to promote systematic use of digital health solutions for health system improvement through better data. The first National Digital Health Strategy followed in 2018, alongside renewed investment in HIS and HMIS by government and partners. Routine health information data are aggregated into HMIS and managed using District Health Information System 2 (DHIS2), an open-source, web-based platform for data collection, management, and analysis. DHIS2 is the world’s largest HMIS platform, used by ministries of health in 76 LMICs, covering 3.2 billion people (about 40% of the global population). Including NGO programs, DHIS2 operates in more than 100 countries (25). Sierra Leone adopted DHIS2 as its national routine health information system in 2008.

Despite these efforts, MOHS has faced persistent challenges in obtaining timely, complete, and reliable data. Health planning and monitoring have often been undermined by inaccurate data, and funds allocated to health programs have been inefficient. Today, disease surveillance and service delivery data are collected through a mix of paper-based and electronic systems. While most community and facility-level data are entered into HMIS, some remain siloed at different levels of care.

In 2019, Sierra Leone introduced a broader National Innovation and Digital Strategy, with a health pillar focused on leveraging big data and AI to improve healthcare overall, particularly maternal and child health (18, 26).

#### 2.3.1 Digital health solutions in primary care and noncommunicable diseases management

The burden of noncommunicable diseases (NCDs), including cardiovascular diseases, cancers, chronic respiratory diseases and diabetes, is disproportionately high in LMICs, accounting for 73% of global NCD deaths (32 million) (27). This escalating burden, intensifying over recent decades, places significant pressure on health systems and reflects an epidemiological transition from infectious diseases to chronic conditions, aggravating existing health challenges (28). Primary care assumes a pivotal role in the prevention, early detection, and management of these chronic conditions. Digital health solutions like mobile health (mHealth) applications, remote monitoring devices, and telemedicine platforms, can bolster primary care providers’ capacity to deliver timely and effective care by monitoring patients’ health status remotely, delivering prompt medical guidance and personalised interventions helping to prevent severe complications (29). Additionally, patients gain better tools and support to manage their own conditions effectively. Digital self-management is an emerging alternative approach to chronic diseases management in Sub-Sahara Africa (30).

Various studies in Sub-Sahara Africa showcase that mHealth solutions can increase disease awareness, promote healthy behavior and provide clinical care. Studies in Nigeria and Cameroon demonstrated that mHealth interventions enabling remote communication between patients, clinicians, and pharmacists (via apps, phone calls, SMS, or voicemail) were associated with improved blood pressure control (31–33). Mobile apps can enable community-based screening for example of people at risk of diabetes and hypertension. By combining a few simple questions with a waist circumference measurement and blood pressure reading, community health workers in Malawi, Sri Lanka and Tanzania can screen for diabetes and hypertension (34). Digital solutions contribute to extending healthcare services to underserved areas and bridge specialist workforce shortages. Remote monitoring using portable ECG technologies allows non-specialist rural health workers to perform ECGs and real-time continuous ECG monitoring using a tablet in remote villages, with recordings transmitted wirelessly via mobile networks to cardiologists in urban centers for interpretation and management guidance. This approach enables early detection of acute coronary syndromes and arrhythmias while improving access to specialized cardiovascular care for rural and underserved populations (33, 35). Overall, digital health solutions can strengthen primary care–led NCD management in low-resource settings by improving prevention, early detection, self-management, and access to specialist care—in order to do so thoughtful considerations of ethical, legal and social issues need to be considered in cultural contexts.

## 3 Ethical, legal, and social issues (ELSI)

There is insufficient suitable infrastructure to support digital health systems in LMICs but also globally (36–40). Poor internet connections, limited or unreliable power supply, outdated technology and lack of data storage can hinder the implementation and effectiveness of digitalisation in medicine (36, 41).

There are ethical, legal and social issues that need to be considered to ensure that the benefits of digitalisation in medicine in LMICs are maximised, and the risks minimised (42, 43). It is important that policy makers, healthcare providers and technology developers work together.

### 3.1 Ethical issues

Digital health solutions must be designed and implemented in a way that maintains or improves the quality of care globally (44). Poorly designed or implemented solutions could have unintended consequences, including misdiagnosis, inappropriate treatment, and harm to patients (41). One of the biggest issues in this setting is lack of interoperability (37, 40), which is even more challenging in LMICs where often multiple non-interoperable digital health systems are used (41, 42). This can lead to fragmentation and duplication of health services and eventually to higher costs, low efficiency and poor patient outcomes.

However, digitalisation in medicine can improve access and healthcare delivery, while also providing insights into disease patterns, healthcare utilisation and other key health indicators (45). It can also improve the potential and efficiency of healthcare services by enabling more accurate diagnoses, better treatment options and better monitoring of patients (46). Furthermore, reduction in waiting times and lower healthcare costs are enabled which can be particularly important in LMICs where resources are limited (47).

#### 3.1.1 Ethical issues: key recommendations for investors

Investors must consider the ethical implications of investing in digital health technologies that are only accessible to those who can afford them and look for ways to improve access to these technologies for all patients, regardless of their socio-economic status. In addition, the ethical implications of investing in technologies that require specialised skills and training should be considered.

### 3.2 Social issues

Use of digital health systems requires proper workforce training and support (39). Given these technologies rely on clinical, technical, and analytical expertise, programmes must build and fund capacity to enable safe, effective, and scalable adoption. Otherwise, the implementation and effectiveness of digital health solutions may be limited. Possible approaches to this challenge could be e-learning for medical education (48), the Basic Emergency Care (BEC) course developed by the WHO in collaboration with the International Committee of the Red Cross (ICRC) to address training gaps in LMICs (36), massive open online courses for health worker education (49) or other programs (50).

Health sector performance is critically dependent on health worker performance because service quality, as well as the accessibility and efficiency of health service delivery, is directly mediated by workers’ ability and willingness to apply themselves to their tasks (51). Resource availability and worker competence are essential but not sufficient to ensure desired worker performance (52). Hence, motivation, which we define as an individual’s degree of willingness to exert and maintain effort toward organisational goals (51), is critical for ensuring that qualified workers perform well. Against this backdrop, performance-based financing has emerged as a promising intervention for improving the delivery of high-quality healthcare services by enhancing health workers’ motivation. Indeed, a qualitative study in Mozambique demonstrated that performance-based financing positively influenced health worker motivation by introducing or reinforcing both internal and external motivational drivers (53).

Digital health technologies can improve the efficiency of healthcare but the context of the application must be carefully assessed to ensure the full benefits are realised. At the same time, care must be taken to ensure that inequalities within population groups are not further exacerbated (54).

LMICs have different cultural practices and beliefs that may influence the use and adoption of digital health technologies (42, 55, 56). Therefore, digital health technologies need to be culturally sensitive and tailored to the needs and expectations of local communities. Digital health technologies may be developed in languages and cultural contexts that are not relevant to LMICs, which can create barriers to adoption and implementation. Cultural and social factors can influence the adoption and effectiveness of digital health solutions. For example, some patients prefer traditional in-person visits with their healthcare providers, while others prefer the convenience of digital healthcare, such as telehealth. Understanding these factors is critical to the successful adoption of digital health solutions.

Digital health technologies have the potential to not only improve access to healthcare and reduce health inequalities in LMICs in general but also improve access to healthcare in remote or underserved areas where traditional healthcare services may not be available. This requires a clear strategy with large investments in these technologies and also investments in healthcare delivery infrastructure (57–59). The goals of investing in health system strengthening are generally to improve the capacity, efficiency, or quality of healthcare delivered, or to expand the range of services offered (60). Many examples show how health workforce training, physical infrastructure, supplies and coordination between healthcare providers can improve health outcomes for individuals at all stages of life (61–63). These supporting infrastructures must not only be built but also maintained. Investment in capacity building is essential to ensure that LMICs can fully benefit from digital health technologies (36, 53, 57–60, 63, 64).

Digitally inclusive health strategies should support patients in using technology at all levels. This includes providing digital skills training, especially for recent adopters or those with devices that have limited functionality (65, 66). Therefore, appropriate regulation is needed to ensure that access to digital health services is not limited to those who have and can afford access to the necessary infrastructure (67). Otherwise, those who do not have access and cannot afford it will be left behind.

Finally, digital tools in LMICs should also be leveraged to build infrastructure for biomedical research, including biobanks. Initiatives such as the Biobank and Population Cohort Network (BCNet) have already set an important precedent (68).

#### 3.2.1 Social issues: key recommendations for investors

Investors need to be aware of cultural differences and ensure that technologies are developed and implemented in ways that respect local norms and practices. They should also consider the broader social impact of their investments. For instance, if a technology requires supporting infrastructure, investors must assess how such infrastructure can be built in LMICs to enable effective adoption. It is equally important to ensure that technology complements rather than replaces human resources, so as not to displace health workers or increase unemployment. Furthermore, investments should focus on technologies that can be realistically implemented and used by health providers, while also creating opportunities to build capacity through training and education.

### 3.3 Legal issues

The digitalisation of medicine generates a huge amount of sensitive patient data, including personal health information, which needs to be protected (69). Ensuring the privacy and security of this data is essential to maintain patient trust, ensure privacy, prevent data breaches, and protect sensitive information from misuse. While blockchain has been proposed as a potential solution, its effectiveness in real-world healthcare settings remains unproven (70).

Digital health technologies are often subject to regulatory requirements to ensure patient safety and privacy (67, 71). In LMICs, these regulations may not be as well established or enforced and may not have the same level of regulatory oversight as in developed countries, leading to concerns about the security and effectiveness of digital health solutions. This may not ensure adequate data protection. As in other parts of the world, patients may also not fully understand the implications of sharing their health data and are therefore unable to give informed consent. However, patients must give informed consent before their personal health data is collected, stored or used in digital health solutions. This requires ensuring that patients understand the potential benefits and risks of digital health solutions, as well as their right to opt out of such solutions. This is particularly important in LMICs where literacy rates and health literacy may be low (42, 56, 68, 69, 72–76).

In many LMICs, there is no adequate legal framework for monitoring digital health solutions. This can lead to a lack of standardisation, quality control and oversight, which can increase the risk of patient harm (42). Investing in digital health technologies can help LMICs adopt international regulations and standards, which can lead to better access to international funding and support (56).

Digital health technologies are often associated with the creation of intellectual property such as patents, trademarks, and copyrights. Intellectual property rights may not be well defined or enforced in LMICs, leading to concerns about who owns and can benefit from digital health solutions (5).

#### 3.3.1 Legal issues: key recommendations for investors

Digital health technologies are associated with risks and investors need to consider the potential liability that may arise from the use of these technologies. These include liability for errors or malfunctions of the technology and liability for breaches of patient privacy.

Investments in digital health technologies often involve contractual agreements with healthcare providers, technology vendors and other stakeholders (77). Investors need to ensure that these agreements in LMICs are legally binding and protect their interests. In LMICs, the legal environment can be less predictable and stable than in high-income countries. Investors need to be aware of the legal risks associated with investing in LMICs, including the risk of expropriation, nationalisation, or other forms of political instability. Investors need to ensure that they have the necessary legal protection to safeguard their intellectual property rights in LMICs.

Investors need to ensure that they invest in technologies that have been proven to work and are centred on evidence-based medicine to provide optimal care for patients. As patient data is collected and shared digitally, data protection laws and regulations must be adhered to, in order to protect patient privacy and avoid legal consequences. Thus, investors must only invest in technologies that prioritise patient privacy and security. Ideally, they must work with healthcare providers to establish appropriate privacy policies.

### 3.4 ELSI: key considerations

Across both LMICs and high-income countries, there is a lack of comprehensive legal frameworks for digital health, including data protection laws, telemedicine regulations, and standards for AI in healthcare. In the absence of clear regulations, there is a risk of exploitation, data breaches, and unaccountable practices. Digital health frequently necessitates the cross-border sharing of data, yet legal ambiguities and incongruent national legislation can impede collaboration and innovation. The efficient and secure exchange of cross-border data is of crucial importance for epidemic response, research, and resource allocation. In the absence of explicit regulations pertaining to data protection laws, telemedicine regulations, and associated domains, there exists a potential vulnerability to exploitation, data breaches, and unaccountable practices.

The FAIR principles (Findable, Accessible, Interoperable, Reusable) are a set of guidelines designed to improve the management and sharing of digital data, especially in research and healthcare contexts. When applied to eHealth in LMICs, these principles have the potential to address some of the most pressing challenges in digital health in these countries, such as data fragmentation, lack of standardisation, and limited access to health information.

Finally, whenever possible, bottom-up strategies should be used to implement eHealth. The involvement of local stakeholders is instrumental in ensuring that the solutions devised are culturally appropriate and are accepted by the end-users. The solutions are customised to align with the specific requirements, linguistic nuances, and operational procedures of each locale, thereby enhancing both the accessibility and the efficacy of the systems. It has been demonstrated that users are more likely to embrace tools they have helped to create, thereby reducing resistance and abandonment. Initiatives that are bottom-up in nature have been demonstrated to be effective in rapidly adapting to changing needs or crises (e.g., Ebola, COVID-19). The local ownership of such initiatives often results in long-term maintenance and a reduced reliance on external funding.

## 4 Financing requirements and challenges

Africa faces an uphill battle in achieving universal health coverage, with various factors such as disease outbreaks, environmental issues, political instability, and security risks impeding. The root causes within the health system are a lack of sufficient investment, capacity limitations, and poor performance across essential building blocks. External challenges include societal, cultural, and physical factors that prevent individuals from accessing healthcare services (78). In response, digital health technologies are increasingly being employed to enhance the delivery of essential health services. By leveraging digital tools, healthcare providers can deliver better patient outcomes, improve access, increase productivity, and lower costs. However, persistent shortcomings remain in coordination, integration, scalability, sustainability, and equitable distribution of digital health investments.

Policy makers must lead efforts to coordinate stakeholders, minimize duplication, direct investments into lagging areas, and ensure long-term funding and sustainability of digital health initiatives (79).

Digital health technologies represent increasingly viable and available solutions to abate the high intensity of healthcare costs by bringing improved efficiencies throughout the value chain. This is especially true in Africa, which accounts for only 1.3% of the global healthcare workforce yet is faced with 25% of the global disease burden (2).

Indeed, a McKinsey & Co white paper concludes that by expanding the use of digital health tools, African health systems could realise up to 15 percent efficiency gains by 2030 (80). Widespread adoption by 2030 could unlock $400 million to $2.5 billion in Kenya (4–14 percent of total projected healthcare spending), $700 million to $3.3 billion in Nigeria (4–10 percent of total projected healthcare spending), and $1.9 billion to $11 billion in South Africa (6–15 percent of total projected healthcare spending); these savings can be reinvested to improve access and outcomes (80).

Despite these opportunities, financing remains a major barrier to digital health scale-up in LMICs. In fact, most African countries have still not achieved the targets set in the 2001 Abuja Declaration, in which governments agreed to allocate 15 percent of their national budgets to health (2). Consequently, the annual health expenditure per capita ($78.7 in 2019) is less than 1/10th of the global average ($1,115.01 in 2019) (81).

As a result, many digital health activities in Africa are funded by (private) development partners leading to the verticalization of digital health services, e.g., the organisation around specific areas of medical practice, such as primary care, mental health, chronic disease management, or telemedicine. This approach can create gaps in sustainability or reach of digital health projects, as investments are determined by partner interests rather than national priorities (82).

Alone, this presents challenges in achieving full coverage, however with effective coordination, horizontal integration of solutions could be achieved. Interoperability of digital technologies is critical, but it is also essential that policy makers, health systems and providers, technology developers and investors work together and identify areas of unmet need (and potential effort duplication) to aid efficiency of resource use. Local pharmaceutical companies should also play a key role, leveraging their networks to bridge these different stakeholders’ groups and facilitate coordination.

To further quantify the problem and need for financing of health systems, the WHO is urging sub-Saharan Africa to raise spending to $271 per capita annually vs. the $189 average from 2023 (83). Representing a > 40% increase over 2023 levels, this increase may aid progress towards achieving SDG 3 within the continent. Towards this target, the African Centres of Disease Control and Prevention (CDC) are championing a New Public Health Order for the continent which entails initiatives and investments across critical areas of policy and practice (84). Digital transformation is a key pillar alongside additional focus areas including, a well-supported workforce; adequate domestic financing; robust technical skills; efficient data management; and reliable regional procurement.

### 4.1 Funding sources

#### 4.1.1 Private funding

Private sector investment in African digital health represents <10% funding for African start-ups. Given the health related challenges facing the African continent, this indicates an underfunded yet high-growth opportunity. Private funding is generally sourced via two key mechanisms, either (1) venture capital or private equity funds (Table 1), or (2) corporate strategic investments (Table 2):

- Venture capital and private equity: The African HealthTech sector has attracted moderate venture capital attention, with $550 million in funding deployed over the past three years, largely driven by online pharmacies, telemedicine and AI-driven diagnostics. Notable investment groups include Vital Capital, Investment Funds for Health in Africa (IFHA), LeapFrog Invesments, Villgro Africa, HealthCap Africa and Jaza Rift Ventures.
- Corporate strategic investment: Major pharmaceutical and technology companies recognise the unmet need and opportunity that presents in Africa and have increased their strategic investments in digital health on the continent. Typically, this is done through private-public partnership such as the Investing in Innovation (i3) program, which has expanded to provide up to $225,000 grants for pharmacy-focused innovations across 19 African countries, supported by Gates Foundation, MSD, Sanofi, and other corporate partners.

**Table 1:** Venture capital & private equity investment funds.

Fund name	Fund size	Target investment areas	Investment stage/range	Geographic focus	Reference
Growth/late-stage funds ($200 M+)					
LeapFrog Investments Fund IV	$1.15 billion	Healthcare & financial services, digital health platforms, diagnostics, pharmaceuticals	$10-50 M (growth stage)	Africa & Asia	LeapFrog Press Release (91) and Adamkiewicz (92)
Vital Capital Fund	$350 million (AUM)	Healthcare infrastructure, water, food security, sustainable infrastructure	$10-30 M	Sub-Saharan Africa	Environmental Finance (93) and ImpactAssets (94)
Norrsken22 African Tech Growth Fund	$205 million	Fintech, edtech, HealthTech/MedTech, market enablement	$2-16 M (Series A/B/C)	Nigeria, Kenya, South Africa, Ghana	TechCrunch (95) and DFC (96)
Novastar Africa Fund II	$200 million	Healthcare, fintech, multiple sectors serving low-income consumers	$250 K-$8 M	Sub-Saharan Africa	Impact Investing Institute (97)
Early/mid-stage funds ($50 M-$200 M)					
TLcom Capital TIDE Africa Fund II	$154 million	Fintech, healthcare, logistics, education, agriculture	$1 M-$3 M initial (Seed/Series A)	Sub-Saharan Africa + Egypt	Kene-Okafor (98) and TLcom Capital (99)
Investment Fund for Health in Africa II (IFHA II)	$137 million	Healthcare infrastructure, hospitals, clinics, pharmaceutical distribution, health insurance	$5 M-$20 M (growth stage)	Sub-Saharan Africa	EIB (100) and FMO (101)
Partech Africa Fund	$124 million	Fintech, digital services, mobility, healthtech	€0.5 M-€5 M	Sub-Saharan Africa	EIB (102)
Al Mada Ventures	$110 million	Fintech, healthtech, logistics, edtech, renewable energy	Growth-stage investments	Pan-African (Morocco-based)	Wamda (103)
Algebra Ventures Fund II	$100 million	Fintech, logistics, healthtech, edtech, agritech	$500 K-$2 M (Seed to Series B)	Egypt, MENA, East/West Africa	Kene-Okafor (104)
Jaza Rift Ventures	$50 million	Medtech, biotech, digital health platforms	$50 K-$500 K (early stage)	Africa (Kenya-based)	Mwangi (105)
Smaller funds (<$50 M)					
Launch Africa Ventures	$36 million	Healthcare, fintech, multiple sectors	Seed to Series A	Pan-African	Launch Africa (106)
4Di Capital	Fund size undisclosed	Fintech, insurtech, healthtech	Early to growth stage	Africa	4Di Capital (107)

Traditional funds seeking financial returns through equity investments; total capital of $2.3B + identified across 13 major funds.

**Table 2:** Corporate strategic investments (equity).

Company/program	Investment type	Investment range	Target areas	Geographic focus	Reference
Health54 (CFAO)	Equity investment	€250 K-€5 M (seed to Series B)	Startups leveraging CFAO networks	Africa	Mwangi (105)
HealthCap Africa	Equity investment	$250 K-$2 M (seed to pre-Series A)	Healthtech & fintech, medical supply logistics, diagnostic tools	Nigeria, Kenya, Egypt, South Africa	Mwangi (105)
Transform Health Fund (AfricInvest)	Debt & mezzanine	Debt & mezzanine financing	Healthcare supply chains, care delivery, digital health solutions	Sub-Saharan Africa	Proparco (108)
Villgro Africa (equity component)	Equity investment	Equity funding (seed stage)	Healthcare innovation, diagnostics, medicine access	East Africa	Mwangi (105)

Companies making equity/debt investments for strategic value (partnerships, market access, supply chain) across 4 major programs.

#### 4.1.2 Public and charitable funding

The public funding landscape has shifted profoundly in 2025, fundamentally reshaping the ecosystem of available capital for global health. The most consequential change is the dissolution of the U.S. Agency for International Development (USAID) in July 2025 (85), removing one of the largest and most reliable sources of U.S. government support for health programmes in low- and middle-income countries (LMICs). For decades, USAID financed a broad portfolio including HIV/AIDS, maternal and child health, and health systems strengthening, meaning its closure has created a substantial funding gap and unprecedented uncertainty for governments, NGOs, and private partners that depended on its support (86, 87).

The full impact of this disruption is still unfolding. In the short term, ongoing projects face delays, downsizing, or abrupt termination, while pipeline initiatives risk cancellation. Over the longer term, the consequences may include slower progress toward universal health coverage, reduced capacity for outbreak preparedness, and heightened vulnerability of digital health programmes that rely on predictable donor funding. This shift also increases pressure on other bilateral and multilateral donors to fill the gap, though their ability to fully compensate remains uncertain, and many national governments face limited fiscal space to expand domestic health spending.

The dissolution of USAID marks a critical inflection point that exposes the fragility of global health financing and highlights the need for more diversified, sustainable funding strategies for digital health in LMICs. Yet public and philanthropic grants continue to represent an essential source of capital, including international aid mechanisms, development funds, and public–private partnerships (Table 3). These instruments typically target areas with pronounced funding gaps, where high risk profiles or limited financial returns deter commercial investors.

**Table 3:** Non-dilutive financing.

Initiative name	Source of funding	Size	Target areas	Funding type	Geographic focus	Reference
Major philanthropic & development commitments						
Gates Foundation Africa Health Innovation Initiative	Bill & Melinda Gates Foundation	$200 billion (20-year commitment)	AI-driven health services, youth innovation, digital health infrastructure	Grants	Africa-wide	Gates Foundation (88)
African Development Bank Health Infrastructure Fund	African Development Bank	$11.1 billion (2024–2025)	Jobs creation, climate action, health systems strengthening, infrastructure	Mixed: Loans + Grants	Africa-wide	Agbetiloye (109)
Horizon Europe Africa Initiative III	European Commission	€500 million	Digital health, AI in biomedical research, pandemic preparedness in partnership with African institutions	Research Grants	Africa-Europe partnerships	EURAXESS (110)
World Bank MADE Alliance: Africa	World Bank Group, AfDB, Mastercard	$300 million (AfDB commitment for first 5 years)	Digital access for 100 million individuals and businesses, agriculture digitization	Mixed: Infrastructure Finance + Grants	Africa-wide	World Bank (111)
EDCTP3 Global Health Partnership	European Commission, EDCTP Association	€214 million (2025)	Tuberculosis, malaria, neglected tropical diseases, clinical infectious disease research	Research Grants	Sub-Saharan Africa	Global Health EDCTP3 (112)
Targeted health programs						
Digital Health Impact Accelerator (DHIA)	The Global Fund, Others	$50 million	Digital health transformation focuses on AIDS, tuberculosis, malaria	Grants	Africa	Global Fund (113)
Smart Africa Digital Academy (SADA)	World Bank, Smart Africa Alliance	$20 million (5-year grant)	Digital skills for 30,000 policymakers and decision makers	Grants	Africa-wide	Smart Africa (114)
Corporate grant programs						
Investing in Innovation (i3)	Gates Foundation, MSD, Sanofi, Cencora, Microsoft, Others	Up to $225 K per grant	Pharmacy supply chain, digital health innovations	Grants	19 African countries	Urban Geekz (115)
eHealth Africa	African Union, Bill & Melinda Gates Foundation	Undisclosed	Health delivery systems, public health emergency management, disease surveillance, laboratory & diagnostic systems	Grants + Technical Assistance	West Africa	eHealth Africa (116)
Villgro Africa (Grant Component)	Villgro Africa	$1.2 million deployed	Healthcare innovation, diagnostics, medicine access, healthcare logistics	Grants + Technical Assistance	East Africa	Villgro Africa (117)
ENDED: Digital Health Ecosystem (DHE)	Bayer Foundation, PATH, Medic	€2 million (2-year initiative from 2022)	Sustainability and expansion of digital tools for community health	Grants	LMICs including Africa	PATH (118)

More than $210B available in grants, loans, technical assistance (no equity taken, focused on development/social outcomes) across 12 major initiatives.

Notably, in June 2025 the Bill & Melinda Gates Foundation announced an unprecedented commitment of approximately 200 billion USD over 20 years with a majority share earmarked for African health innovation (88). This is widely recognised as the largest single philanthropic pledge to continental development in modern history. This long-term commitment prioritises AI-enabled health services, youth-led innovation, and digital health infrastructure, with a strong focus on strengthening health systems and technology platforms across Africa.

It should be noted that the mapping of major public and charitable funders supporting digital health initiatives in sub-Saharan Africa presented here is based on targeted searches of publicly available information. As such, it may not fully capture the breadth or rapid evolution of this funding landscape.

### 4.2 Key success factors for investment

To encourage investment and maximise success, it is very important that entrepreneurs receive not only financing but also adequate in-kind support from governments, industry and institutional players. This includes access to technical and operational expertise, supportive infrastructure, and appropriate fiscal or policy incentives, alongside other emerging forms of support such as regulatory alignment and AI-focused capacity-building initiatives (as evidenced by initiatives from the Gates Foundation) (89).

Digital health initiatives range in size and scope, and source from both African and non-African based organisations. This fragments the funding and reaffirms the need for each country to develop their own strategy to optimise capital access and use and to ensure equitable distribution throughout the value chain.

Digital health investments compete for limited resources given other urgent health priorities (clinics, vaccinations, medicines, workforce), making it essential to demonstrate clear value and impact. This underscores the critical importance of robust impact appraisal technique, so that digital health investments deliver measurable value even under constraints and incomplete data (90). Instead, effective investment approaches in the African context prioritize flexible funding models, strong local partnerships, and integration with existing health system data streams. Rather than relying on external evaluations or rigid milestone payments, successful digital health initiatives achieve accountability through co-designed governance structures, sustained technical support, and long-term collaboration between funders, governments, and implementing partners.

## 5 Conclusion

### 5.1 Discussion

Digital solutions can help address healthcare access and efficiency challenges in LMICs, particularly in sub-Saharan Africa, where mobile connectivity is expanding and mHealth platforms are increasingly deployed for disease surveillance and management. The rapid evolution of digital health innovation in the region demonstrates growing momentum, with forty-one out of fifty-four African countries now having national digital health strategies.

However, digital health investments must be embedded in robust governance frameworks and aligned with national priorities to avoid the fragmentation and duplication that has characterized previous efforts. The main text illustrates through examples such as Sierra Leone’s experience during the Ebola response how poorly coordinated digital health interventions create short-lived projects with minimal evidence, and how moving from fragmented emergency responses to coordinated national strategies requires clear governance structures and strategic planning.

Workforce training, patient education, and cultural context are critical factors in determining whether digital health solutions are adopted and sustained. The text emphasizes that cultural differences between and within LMICs require tailored approaches rather than one-size-fits-all solutions, and that both healthcare workers and patients require proper training and support. Additionally, interoperability and data standardization are essential to leverage existing infrastructure cost-effectively and enable collaboration between national and international systems.

Long-term financing and coordination are essential for sustainable implementation. Digital health investments must be defined and implemented according to local, national, and supranational needs, with clear prioritization to prevent duplication and ensure alignment with broader health system objectives.

### 5.2 Limitations

The present paper constitutes a concise synopsis of several pivotal aspects pertaining to the phenomenon of digitalisation within the context of LMICs, as these aspects have been observed and experienced in our day-to-day professional practice. This is an opinion paper based on the authors’ applied knowledge, supplemented by additional literature and funding research, and does not provide an exhaustive assessment of digital health initiatives in LMICs. The focus of the paper is on countries in sub-Saharan Africa, with the intention of geographically delimiting the LMIC. However, the validity of the aforementioned argument is not exclusive to this region; it is applicable to a considerable proportion of LMICs in disparate geographic areas.

It was not possible to address every element in a comprehensive manner, including both favourable and unfavourable aspects. Digital transformation is occurring at an unprecedented rate, rendering the relevance of current concepts and methodologies highly uncertain in the future. It is therefore hoped that the present paper will encourage other experts to share their experiences. The present opinion paper is constrained by limitations of space, which preclude discussion of several significant issues. These include, but are not limited to, the following: cybersecurity and data governance concerns; the environmental impact of digital infrastructure; gender disparities in digital access and literacy; the role of local and indigenous innovation versus imported solutions; and access for persons with digital literacy.

The authors acknowledge that the financing section draws on funding commitments intended for future investment and recognise that, in a rapidly evolving context, both allocations and priorities are subject to change. Moreover, volatility in the funding environment may limit the long-term accuracy and relevance of the funding landscape assessment.

Digitalisation in medicine is profoundly altering the manner in which medical care is delivered in LMICs. It is anticipated that advancements in the field of digitalisation will positively impact the realm of medicine within LMICs, albeit with the possibility of intermittent setbacks.
